# Rock River Union Medical Society

**Published:** 1866-03

**Authors:** Oliver Everett, H. C. Donaldson

**Affiliations:** President; Secretary


### ROCK RIVER UNION MEDICAL SOCIETY.

The Rock River Union Medical Society held its semi-annual meeting at Dixon, Ill., Dec. 13th, 1865.

In absence of the President and Vice-President, Dr. O. Everett, of Dixon, was elected to the chair.

The minutes of the last meeting were read and approved.

Dr. Henry E. Paine and Dr. I. S. Williams, of Dixon, were admitted to membership.

Dr. Phillips, of Dixon, presented a specimen of granular disease of the kidney, with remarks.

Dr. Paine, of Dixon, presented a specimen of congenital imperforate anus, with the following history:

Mrs. W. gave birth to a male child, May 5, 1865, primipara. The child was born before the physician in attendance arrived, consequently, the body of the infant was not inspected, and the real condition of the child was unknown until May 6th. When seen, May 6th, the child presented a good development. The skin was jaundiced; he had been fretful for twenty-four hours previous; had urinated frequently; nursed very little. On examining the fundament, I found that no anus existed. The tissues were hard, and as solid at the point where the rectum should have terminated as at any portion of the gluteal region; therefore, I believed that the rectum terminated high up, and that, in order to operate successfully, a very deep incision and dissection would be necessary, and that the prognosis was bad, both as far as the operation and the ultimate result were concerned. While examining the case, and placing the subject in proper position for the performance of the operation, the child expelled its urine, which was tinged with meconium. The existence of a communication between the rectum and urinary organs was thus demonstrated, and though the character of the channel could not be comprehended, and the nature of the deformity was made more complex, yet I determined to make an effort to reach the intestine and relieve the parts. The operation was performed in the usual manner, and an incision carried one and a half inches into the pelvis; little blood was lost; the intestine was not reached, and after a careful and patient exploration, further interference was desisted from. The child died two days after, and I made a post mortem examination. The abdomen was greatly enlarged and tympanitic. On opening the abdominal cavity, a large amount of serum was found, omentum congested, intestines distended with flatus. On removing the urinary organs, kidneys and bladder, together with the rectum, the following condition of organs was found: The bladder was empty, and both ureters were greatly distended with urine, and especially the left, which was as large as a pipe-stem, and filled with urine. The left kidney had undergone cystic degeneration, and was diminished in size to that of a body $\frac{3}{4}$ of an inch long by $\frac{3}{8}$ inch wide. The cyst contained about six drachms of fluid. The right kidney was apparently healthy. On tracing the rectum downward, I found it terminated in a sinus, whose calibre was sufficient to allow a large straw to be introduced; it was $1\frac{3}{4}$ inches long, and communicated with the urethra just anterior to the sphincter muscle of the bladder.

Several interesting cases of resection were reported by Dr. Abbott and Dr. Paine, of Dixon, performed in the army.

Dr. Abbott reported a case of gall-stones, in an old man supposed to have cancer of the stomach, as there was regurgitation of food after eating; but a post mortem revealed the presence of eight or ten gall-stones in a cyst formed by an enlarged uriniferous tube. The duodenum was contracted from deposit of calcareous matter in the coats of the bowel.

On motion of Dr. Phillips, a committee was appointed to prepare a report to be published in the Dixon, Morrison and Sterling papers, preparatory to the anticipated appearance of cholera in our midst the coming season, suggestive of measures both hygienic and sanitary calculated to prevent the spread of the disease.

The Chair appointed as said committee: Drs. Phillips and Paine, of Dixon; Dr. Hagey, of Sterling; and Drs. Nowlan and Donaldson, of Morrison.

Drs. Abbott and Donaldson were appointed delegates to the State Medical Society.

Drs. Everett and Winn, delegates to the National Medical Society.

The Society voted that the Secretary forward these minutes to both medical journals of Chicago for publication.

Adjourned to meet at Morrison, the second Wednesday in June next. OLIVER EVERETT, *President*.

H. C. DONALDSON, *Secretary*.

